# Nature or nurture in mosquito resistance to malaria?

**DOI:** 10.1016/j.pt.2007.01.011

**Published:** 2007-04

**Authors:** Hilary Hurd

**Affiliations:** Institute of Science and Technology in Medicine, Centre for Applied Entomology and Parasitology, Huxley Building, Keele University, Keele, Staffordshire, ST5 5BG, UK

## Abstract

The genetic basis of mosquito resistance to malaria parasites is well established and currently receives a lot of attention. However this is not the sole determinant of the success or failure of an infection. In a recent article, Lambrechts and colleagues report the influence of the quality of the external environment of a mosquito on infection. They indicate that external variations could substantially reduce the importance of resistance genes in determining infection by malaria parasites. Furthermore, these variations could influence future plans to use malaria-resistant transgenic mosquitoes to control parasite transmission.

## The genetic basis for resistance to malaria

Host traits for parasite resistance are usually regarded as being heritable. This premise holds true for several specific associations between species of *Plasmodium* and their mosquito vectors. It is possible to select for increased resistance within the laboratory environment [1–5] and studies have now identified changes in the transcription of several mosquito genes in response to infection with malaria parasites [6]. A recent article by Lambrechts *et al.* [7] challenges this current trend to focus solely on the genetic basis of resistance to malaria. Lambrechts *et al.* explored the influence of environmental quality on the genetic component that underlies the burden and intensity of malaria parasite infection. By doing this, they revisit the nature–nurture debate in the important context of malaria transmission. Their findings, although limited, should stimulate more research to aid in the understanding of the coevolution of mosquito–malaria associations. It should also help researchers in the evaluation of genetic engineering of noncompetent mosquitoes as a viable strategy for malaria control.

## Investigating the importance of the environment

The starting point for the study by Lambrechts and colleagues [7] is the observation that the genetic component of resistance to infection can be influenced by differences in the environment in which those genes are expressed. Furthermore, if a host response to infection is more greatly influenced by environmental factors than inherent resistance mechanisms, the effect of resistance alleles could be masked. They hypothesize that natural populations of vectors will experience differences in environmental quality within and between locations. These differences could influence both epidemiology and host–parasite coevolution and, thus, outcomes could vary in different locations.

Lambrechts and colleagues initiated an investigation of the effect of the environment on parasite resistance using the malaria vector *Anopheles stephensi* and the rodent malaria parasite *Plasmodium yoelii yoelii*. Although *A. stephensi* is not a natural host for *P. y. yoelii*, and would thus shed little light on the coevolutionary aspects of resistance, it is a commonly used laboratory model. Resistance to *P. y. yoelii* infection was determined by counting the presence or absence of developing oocysts on the midgut (prevalence of infection) and the number of oocysts present (intensity of infection).

Variation in the environment was provided by feeding adult mosquitoes different concentrations of glucose solutions after an initial, infective blood meal. The authors had previously shown that the response of one aspect of the defence response of *A. stephensi,* namely encapsulation of a Sephadex® (Sigma Aldrich: http://www.sigmaaldrich.com) bead with melanin, increased with the provision of increasing sugar concentration following a blood meal [8]. Interestingly, a laboratory colony of *Anopheles gambiae* that was fed on high sugar concentrations was able to melanize beads even without a blood meal [9]. This indicates that the effect of the environment might differ between mosquito species.

An isofemale line is an inbred line of mosquitoes that have been derived from the progeny of one female. In this experiment, isofemale lines were created from eight female *A. stephensi* mosquitoes, with descendents maintained for four generations. Each line was then given 2%, 4% or 6% glucose solutions and mosquitoes were then fed on gametocytaemic mice (i.e. those that were known to be carrying gametocytes) four to five days post emergence [7].

## A genetic component

Lambrechts *et al.* found no difference in infection prevalence between these eight lines. Overall, only 12% of mosquitoes were uninfected when examined eight days post infection; therefore, only a small number of mosquitoes were able to eliminate all oocysts. In future studies, it would be interesting to examine salivary glands for sporozoite infections because resistance mechanisms might operate at the sporozoite stage and, thus, further differences between lines could have been missed in this study [7].

Intensity of infection did, however, differ significantly between lines, with median values ranging from three to 26 oocysts per line. Medley *et al*. [10], using several malaria–mosquito associations, showed that intensity and prevalence are predictably related. On the basis of this finding [10], it is surprising that prevalence does not also differ between lines; however, this might not be apparent because of the small sample sizes used – typically, samples sizes of >50 mosquitoes are recommended [10]. Although Lambrechts and colleagues conclude that resistance to malaria parasites by *A. stephensi* has a genetic basis, they suggest that the genetic component that governs prevalence and intensity might differ. Because prevalence is determined by the ability (or failure) to eliminate all malaria parasites that have been taken up during an infectious blood meal, any differences between the genetic components that are responsible for governing prevalence, as opposed to intensity of infection resistance, would be contingent on the capacity of the products of one or more genes to result in the killing of all parasites, rather than many or most parasites.

## An environmental influence on infection

**Figure 1:**
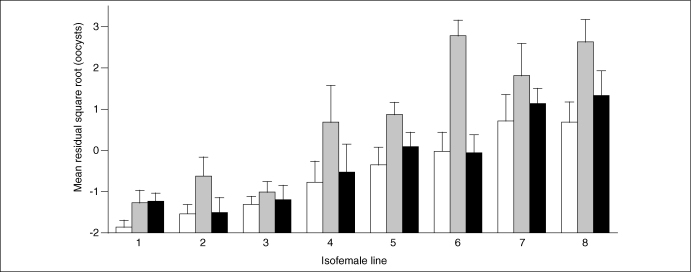
Genetic and environmental components of infection load. The mean number of oocysts (plus standard error) in infected mosquitoes is given for eight isofemale lines that were fed on 2% (white bars), 4% (grey bars) or 6% (black bars) glucose solutions. The lines are ranked along the *x*-axis according to their mean number of oocysts (averaged across glucose concentrations). The number of oocysts has been transformed using the square root and corrected with regards to the mice that were used to feed the mosquitoes (the residual gives the difference from the average for a given mouse). Reproduced, with permission, from Ref. [7].

Lambrechts *et al.* found that the number of oocysts was significantly altered by the richness of the environment. The 4% glucose feed resulted in mean values of approximately twice as many oocysts compared with other feeding regimes, whereas the highest glucose concentration did not differ significantly from the lowest (2% = 11.4 oocysts per midgut, 4% = 22.5, 6% = 13.3) (Figure 1). Although this pattern was observed in all isofemale lines, considerable variation in the degree of the effect of glucose concentration did occur. The authors offer two explanations for this increase and decrease in oocyst burden with increasing sugar concentration. First, based on their previous work [8], they suggest that greater nutritional input might enhance the immune response directed against the parasite. If correct, this could explain the lower infection burden following a 6% feed compared with a 4% feed. It is certainly true that deployment of resistance mechanisms, such as melanotic encapsulation and antimicrobial peptide production, is costly to the mosquito [11]; therefore, the immune response could be limited by available resources. Second, they explain the lower parasite burden after a 2% glucose feed by making the observation (which is still controversial [12]) that high parasite loads cause mosquito mortality. They indicate that females with the highest infection load in the resource-poor sugar meal group die; hence, those surviving would have fewer oocysts than the 4% sugar group.

## Genotype-by-environment interactions

The effect of the 4% feed was seen in every isofemale line but the influence of this environmental indicator on phenotypic variation differed according to line, with the greatest increase seen in lines two and eight. Although glucose concentration effects were detected, genotype-by-environment interactions were not significant. It is interesting to note that the effect of the environment on resistance would not have been detected had the experiment been conducted just with the lowest or highest sugar concentration. This highlights the way that resistance can change according to fluctuations in environmental conditions.

The environmental element did not have a major effect on oocyst intensity because it contributed to only 11.7% of phenotypic variance compared with 35.4% for the genetic component. However, as the authors point out, this is only one of many environmental factors that could have been tested. Indeed, environmental influences could be synergistic. Although female mosquitoes can feed on plant-sugar sources [13,14], these sugar meals might not be important to mosquito populations in the field that have regular access to blood meals [15].

There is clearly a need to investigate interactions between resistance genotypes and other environmental factors that could be as, or more, important in the field as sugar meal concentrations. These could include temperature fluctuations, which have been shown to impinge of the background genetic basis of host resistance [16]. Even such influences as the distance either from oviposition sites or from future blood meals and the presence of potential predators will alter metabolic resources devoted to flight and could, therefore, change the outcome of resistance to infection.

## An environmental determinant of mortality

There is still considerable controversy that surrounds the effect of *Plasmodium* infection on mosquito mortality [12]. Lambrechts *et al.* [7] provide firm evidence in support of the negative impact of infection on mosquito fitness. All eight isofemale lines, whatever their environmental regime, suffered increased mortality during the eight days after an infected blood meal compared with similar groups that were fed on uninfected blood. Unsurprisingly, most mortality was associated with the lowest sugar concentration and it would be interesting to see whether any of these resource-starved mosquitoes survived long enough to sustain salivary gland infections and, therefore, contribute to malaria transmission and indeed whether this finding holds true for natural mosquito–malaria combinations.

## Implications for the field: the way forward

Laboratory populations of mosquitoes are highly inbred and likely to exhibit many biological aberrations compared with wild populations [17,18]. In view of the findings of Lambrechts and colleagues [7], it is now important to assess not only the effect of environmental influences that could be relevant in the field or semifield (i.e. a large outside enclosure) situation but also to do so with natural populations of mosquitoes and *Plasmodium*. Furthermore, it is possible that findings from field studies will only be relevant for the particular area of study. In the wild, different populations encounter different environmental influences – could they also respond differently to them?

## Perspective

Lambrechts and colleagues have shown that the environment matters for the functioning of a malaria-resistant phenotype in mosquitoes [7]. They report an important proof of principal that could have profound effects on the dynamics and coevolution of this vector-borne parasite. Resistance phenotypes are complex, even in laboratory strains, and it remains to be seen whether nurture has a substantial affect on malaria transmission in the field.
